# Ischemic stroke protected by ISO-1 inhibition of apoptosis via mitochondrial pathway

**DOI:** 10.1038/s41598-023-29907-z

**Published:** 2023-02-16

**Authors:** Wanli Ji, Yaoxin Ren, Xiaolian Wei, Xiangxiang Ding, Yihan Dong, Bin Yuan

**Affiliations:** 1 Department of Neurology II, The First Affiliated Hospital of Xinxiang Medical University, Xinxiang Medical University, 88 Jiankang Road, Weihui, Xinxiang, 453100 Henan China; 2 Henan Key Laboratory of Neural Regeneration, Weihui, Xinxiang, 453100 China

**Keywords:** Biomarkers, Medical research, Neurology

## Abstract

Macrophage migration inhibitory factor (MIF) is an immune mediator associated with inflammation, which is upregulated after ischemia in brain tissue. ISO-1 is a potent inhibitor of MIF tautomerase and can protect neurons by reducing the permeability of blood brain barrier (BBB). In this study, we investigated the role of ISO-1 in cerebral ischemia/reperfusion injury by establishing a model of middle cerebral artery occlusion/reperfusion in rats. Rats were randomly divided into four groups: the sham operation group, the ISO-1group, the cerebral I/R group, and the ISO-1 + I/R group. We assessed the degree of neurological deficit in each group and measured the volume of cerebral infarction. We detected the expression of MIF in the core necrotic area and penumbra. We detected the expression of apoptosis-related proteins, apoptosis-inducing factor (AIF), endonuclease G (EndoG) and cytochrome c oxidase-IV (COX-IV) in the ischemic penumbra region. The results showed that MIF was expressed in the ischemic penumbra, while the injection of ISO-1 was able to alleviate neurological damage and reduce the infarction volume. In the cerebral ischemic penumbra region, ISO-1 could reduce the expression of Bax and Caspase3 and inhibit the displacement of AIF and EndoG to the nucleus simultaneously. Besides, ISO-1 also exhibited the ability to reduce apoptosis. In summary, ISO-1 may inhibit neuronal apoptosis through the endogenous mitochondrial pathway and reduce the injury of brain I/R after ischemic stroke.

## Introduction

Ischemic stroke was one of the most common types of stroke, which seriously damaged human health^1,2^. After the cerebral ischemia happened, neurons in the ischemic core area was irreversibly damaged immediately, as well as a reversible ischemic penumbra formed around the ischemic core area, which would shrink gradually as time went by. An important way to rescue neurons in the ischemic penumbra area was the reconstruction of cerebral blood flow. However, the brain tissue in state of long-term ischemia and hypoxia might be injured in the process of artery recanalization, which was called cerebral I/R injury^3,4^.

Various cell death programs could be activated by cerebral I/R injury, such as necrosis and apoptosis^5,6^. Among them, apoptosis was considered a key event in ischemic brain injury^5^. Apoptosis, a programmed cell death was prevalent in penumbra areas after cerebral I/R, and has attracted much attention in the treatment of ischemic stroke due to its reversibility^7^. Mitochondria were the power source of cells and played a key role in determining cell death^8,9^. When cerebral I/R breaks balance, mitochondrial AIFs were transferred to the nucleus, which led mitochondrial dysfunction and promoted apoptosis eventually^10,11^. Previous study had proved that inhibition of AIF transfer was able to inhibit apoptosis^7^, which provided a new method to protect neurons.

MIF was a kind of pleiotropic protein that could be expressed in a variety of cells, especially neuron. Apart from that, it could act as a neuromodulator in neurons^12^. It was reported that MIF was upregulated after cerebral ischemia^13^, and knockdown of MIF could reduce the cerebral infarct area in mouse^14^, which suggested MIF played a deleterious role in the process of cerebral ischemia. ISO-1 was a potent inhibitor of MIF tautomerase and offered protection against many neurological diseases, such as Alzheimer's disease^15^, diffuse axonal injury^16^ and so on. It was also be found that ISO-1 could protect neurons by reducing the permeability of BBB^17^.

In this experiment, we observed that the MIF inhibitor ISO-1 reduced the transfer of apoptosis-inducing factor (AIF) and EndoG, which was from mitochondria to the nucleus, in nerve cells and investigated whether the mitochondrial pathway was involved in the protective effect of ISO-1 on ischemia–reperfusion injury.

## Materials and methods

### Animals

Thirty-six healthy SPF adult male SD rats(6–7 weeks old, 230–260 g) were fed under the condition of room temperature (23 ± 1 °C) and 12 h of alternating light and dark cycles, and were free to obtain food and water. The rats were provided by Jinan Pengyue Experimental Animal Breeding Co., Ltd. Rats were randomized into four groups: the sham operation group, the ISO-1group, the cerebral I/R group, and the ISO-1 + I/R group. This experiment complied with the provisions of the Guide to Experimental Animals of the People's Republic of China and had been approved by the Animal Ethics Committee of the First Clinical College of Xinxiang Medical University (2020026). This study followed ARRIVE animal reporting guidelines.

### Cerebral I/R models

The cerebral ischemia–reperfusion model was made by line bolt method, and the specific process was based on methods of Yuan Yang et al.^18^, and was improved according to our case. Rats were anesthetized with 2% pentobarbital (2 ml/kg), then the left common carotid, external carotid and internal carotid artery were isolated and ligated at the vascular bifurcation. A line bolt was removed after 2 h ischemia and reperfusion lasted for 24 h. No line bolt was inserted in the sham group, and no surgical procedure was performed in the ISO-1 group.

### Pharmacological treatment

When the line bolt was removed, ISO-1 was (Selleck Chemicals, Houston, Texas, USA) dissolved in dinitrosulfoxide (DMSO) at a concentration of 1 mg/kg and injected intraperitoneally. Sham and I/R group were injected with the same dose of DMSO.

### Behavioral tests

Neurological deficit was assessed using the modified neurological severity score (mNSS)^19^ after 24 h brain reperfusion. The score mainly measured motor ability, sensory, and reflex function. The highest score was 11, and the higher score represented heavier degree of neurological deficit in the rats.

### Infarct volume measurement

The brain tissues were cut into six 2 mm-thick coronal sections and incubated in 2%TTC (Solarbio, Beijing, China) at 37 °C for 30 min. After that, slices were fixed in 4% neutral formaldehyde and photographed on the next day. Pictures were analyzed using ImageJ software (NIH, USA).

### Nissl staining

Rat brain tissues were fixed with 4% neutral formaldehyde for 48 h, then embedded in paraffin and cut into 3 μm-thick slices. Slices were dewaxed in xylene and dehydrated in graded ethanol. The slices were stained with Nissl (Solarbio, Beijing, China) dye solution. The changes of Nissl bodies and the pathological morphology of brain tissues were observed under light microscope (55i, Nikon, Japan).

### Immunohistochemistry

Brain slices were dewaxed and rehydrated, put into a heat-resistant glass container containing sodium citrate buffer, heated in a microwave oven for 10 min for antigen repair, and then were permeabilized in 3% hydrogen peroxide and 0.2%Triton buffer for 10 min. Between permeabilization procedures, slices were washed in PBS for 5 min and the wash was repeated 3 times. The slices were blocked with 5% fetal bovine serum for 30 min and then cultured overnight at 4 °C with Rabbit Anti-MIF Polyclonal Antibody (1:200, Abbkine, Wuhan, China). Next day, HRP labeled Goat anti-rabbit IgG was used for incubation for 1 h. Sealed slices with neutral gum. MIF expression in the infarct core and penumbra zone were observed under light microscope.

### Western blotting

Cerebral ischemic penumbra tissues were collected from different groups according to the method of Stephen Ashwal et al.^20^. RIPA containing protease inhibitor was added to brain tissues and the brain tissues were homogenized in a glass homogenizer. Brain protein samples were isolated by 15% polyacrylamide gel electrophoresis and then transferred to PVDF membrane (Millipore, Shanghai, China). The membrane was blocked with 5% nonfat milk powder for 2 h. Primary antibody Rabbit Anti-MIF Polyclonal Antibody (1:1000, Abbkine, Wuhan, China), Rabbit Anti-Bax Antibody (1:1000, Wanleibio, Shenyang, China), Rabbit Anti-Bcl2 Antibody (1:500, Wanleibio, Shenyang, China), Rabbit Anti-caspase3 Antibody (1:1000, Wanleibio, Shenyang, China), Rabbit Anti-cytochrome c oxidase-IV Antibody (1:1000, Affinity Biosciences, Beijing, China), Rabbit Anti-AIF Polyclonal Antibody (1:1000, Bioss, Beijing, China) were added, and stayed overnight at 4 °C. Next day, the membrane was washed three times in TBST for 10 min. Later, HRP labeled Goat anti-rabbit IgG or HRP labeled Goat anti-mouse IgG (1:5000, Epizyme Biotech, Shanghai, China) were used for incubation for 1 h. The samples were washed with TBST again and were added with hypersensitive immunoblot luminescent solution. In order to obtain better gel images, we cut the PVDF membrane before incubating the antibody and only the part of the target proteins was retained. Images were photographed with a fluorescence imager (Imager600, Amersham, USA) and subsequently analyzed with ImageJ software.

### Terminal deoxynucleotide-transferase (TdT-)-mediated d-UTP nicked end labeling (TUNEL) staining

After brain slices were dewaxed and rewatered, 100 μl of protease K working solution was added to each tissue slice, which was incubated in 37 °C oven for 30 min and washed with PBS 3 times and 5 min each time. TUNEL reaction mixture (Keygen Biotech, Jiangsu, China) was added on tissue. Slices were put in a wet box to avoid light and incubated in oven at 37 °C for 1 h. Apoptotic cells were dyed red. Slices were sealed with anti-fluorescence quenching agents containing DAPI. Finally, apoptosis of brain tissue was observed by fluorescence microscope (DM4B, Leica, Germany) and photographed for subsequent analysis.

### Immunofluorescence

After the TUNEL reaction, brain slices were washed 3 times with PBS and 5 min each time. Primary antibody Rabbit Anti-AIF Polyclonal Antibody (1:100, Bioss, Beijing, China), Rabbit Anti-ENDOG Polyclonal Antibody (1:25, Signalway Antibody, USA) were added, and slices were left overnight at 4 °C. Next day, slices were incubated with FITC-labeled goat anti-rabbit IgG (1:100, Biosharp, Anhui, China) for 1 h. Finally, slices were sealed with anti-fluorescence quenching agents containing DAPI and brain tissue was visualized by fluorescence microscopy.

### Statistical analysis

All data in this study were expressed with mean ± standard mean error (SEM) and analyzed using Graphpad Prism 8.0.1 software. Difference between multiple groups was analyzed using one-way analysis of variance (ANOVA), and data between two groups were analyzed using unpaired t-test. The difference was statistically significant when *P* < 0.05.

## Results

### The MIF inhibitor ISO-1 reduces the mNSS score

**Figure 1:**
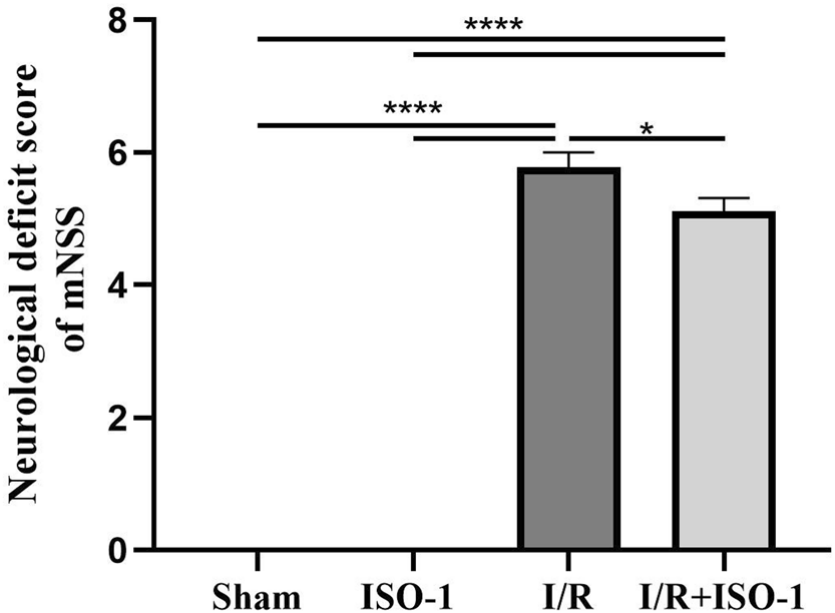
Behavioral assessment was performed by the mNSS test. Compared with the I/R group, scores were lower in the I/R + ISO-1 group. *P < 0.05.

There were no symptoms of neurological deficit in the Sham and ISO-1 groups, which was recorded as 0 points. Groups I/R and I/R + ISO-1 had symptoms of neurological deficit, and the I/R + ISO-1 group had lower neurological severity scores than the I/R group (*P* = 0.0176, unpaired t-test) (Fig. 1).

### The MIF inhibitor ISO-1 reduces the infarct volume

**Figure 2:**
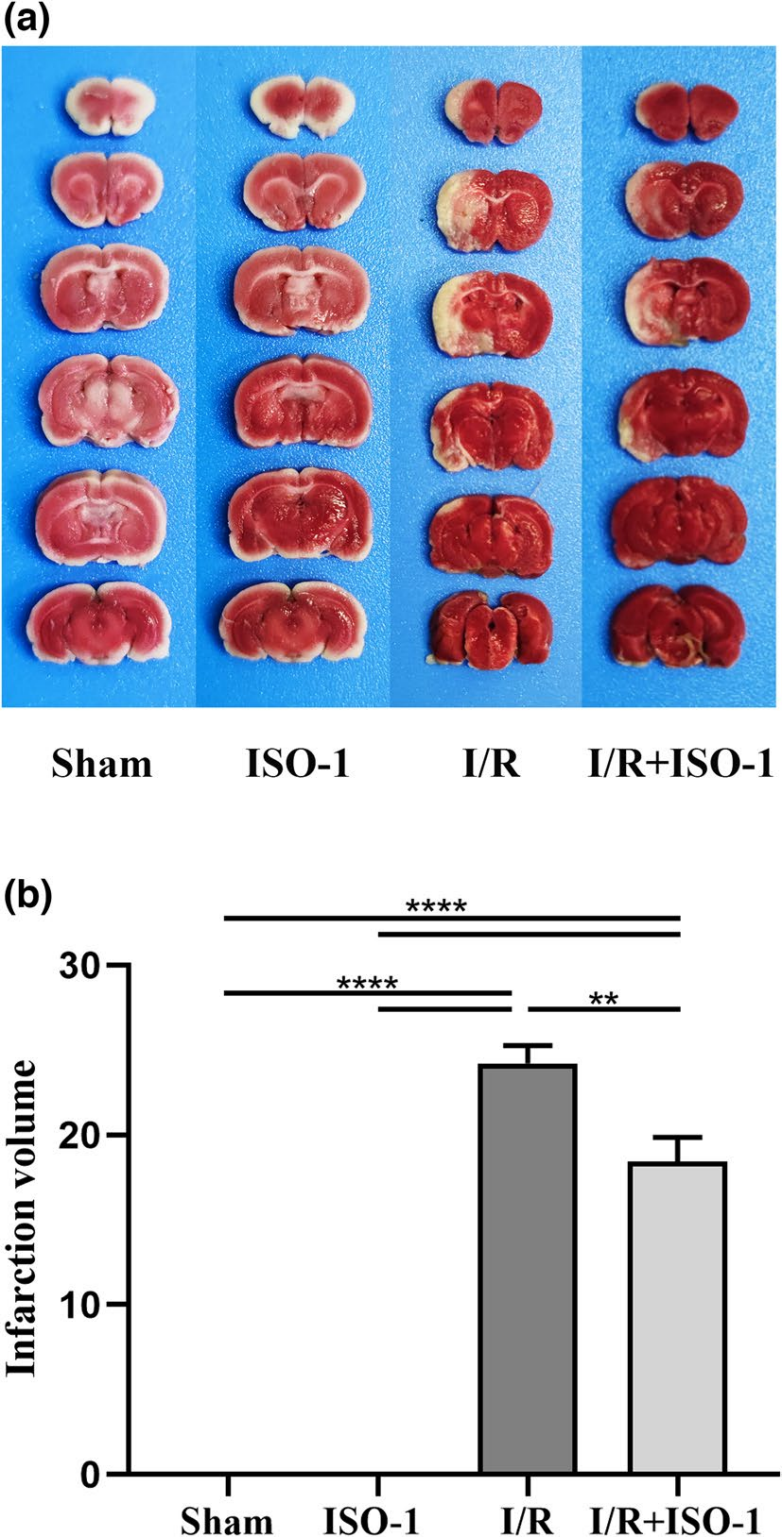
Effect of the MIF inhibitor ISO-1 on infarct volume after 2 h cerebral ischemia and 24 h reperfusion in rats. (**a**) TTC staining in each group, with normal brain tissue in bright red and infarcted tissue in white. (**b**) Quantitative analysis of the infarct volume in each group. **P < 0.01, ****P < 0.0001.

There was no infarct in the sham and ISO-1 groups, while infarct could be observed as white tissue in the I/R and I/R + ISO-1 groups. What’s more, the volume of white infarct part of I/R + ISO-1 group was significantly smaller than that in I/R group (*P* = 0.0071, unpaired t-test) (Fig. 2).

### The MIF inhibitor IOS-1 increases the number of Nissl bodies in the penumbra zone

**Figure 3:**
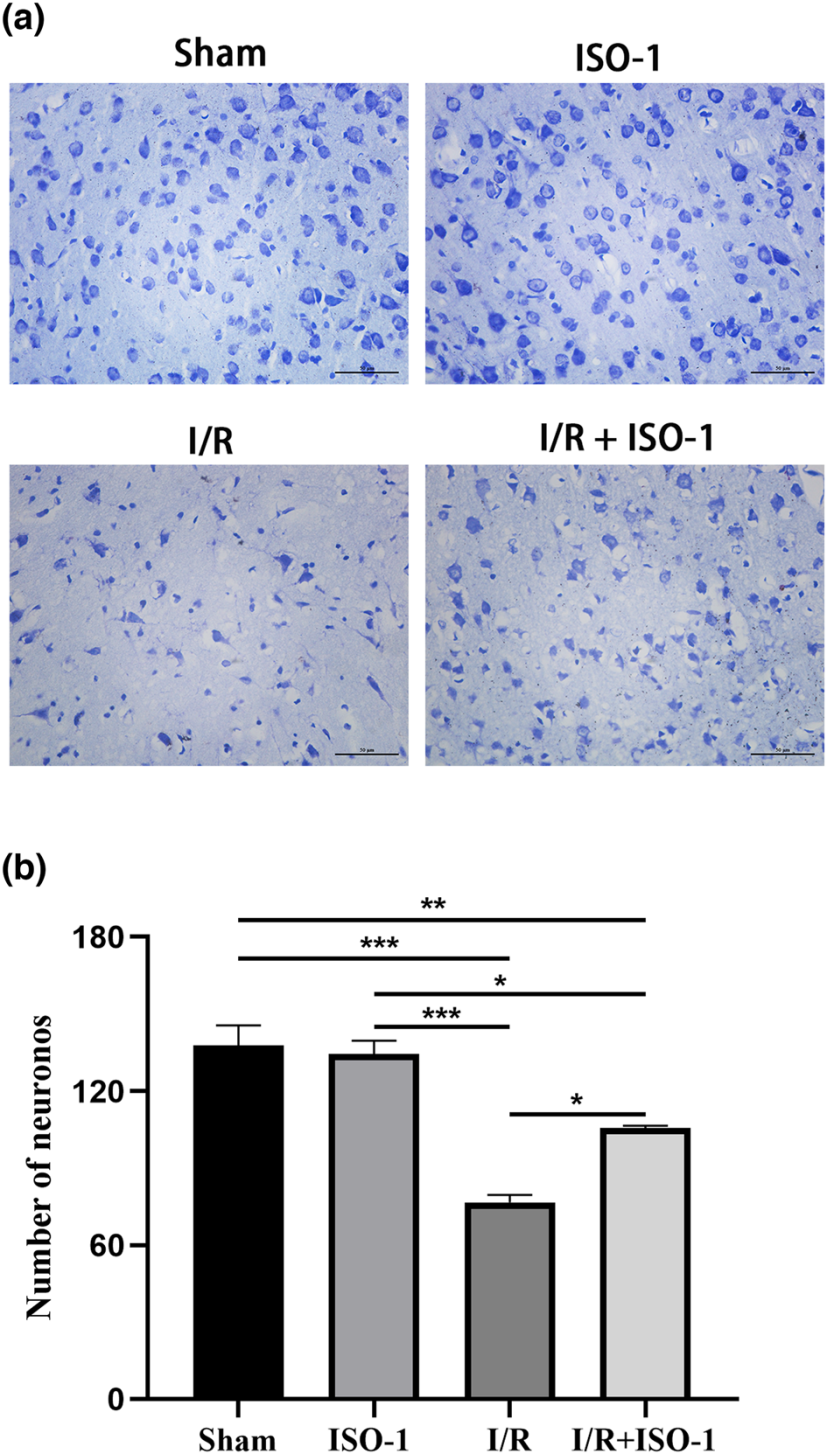
ISO-1 treatment rescued neuronal death after ischemia–reperfusion. (**a**) Representative micrographs of Nissl staining. Bar 50 μm. (**b**) Quantitation of Nissl-stained neurons. *P < 0.05, **P < 0.01, ***P < 0.001.

The result of Nissl staining showed that the tissues in the sham and ISO-1 groups were normal, presenting as a round shape, loose cytosolic pattern and uniform distribution. In group I/R, we observed loose tissues, swelling neuronal cell bodies and nucleolar deviation and Nissl staining showed long-spindle shaped Nissl bodies appeared as scattered distribution with more shallow color, smaller size and decreased number. Compared with the I/R group, more complete and denser brain tissue around ischemia, more neurons and a partial recovery of Nissl bodies were observed in the I/R + ISO-1 group (*F* = 32.55, *P* = 0.0145) (Fig. 3).

### The MIF inhibitor ISO-1 inhibits MIF expression in the penumbra region

**Figure 4:**
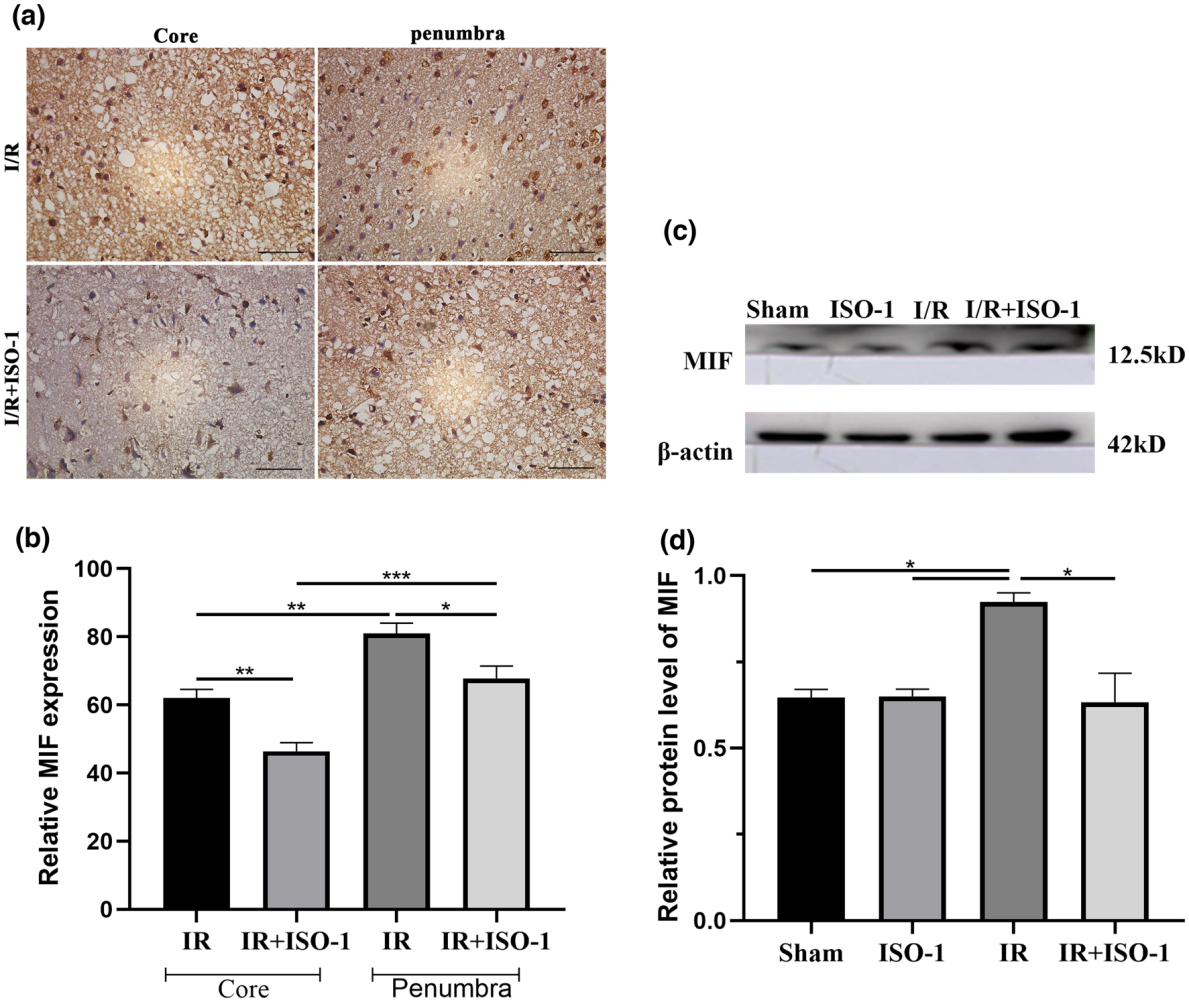
Western blot and immunohistochemistry of the expression of MIF. (**a**) Immunostaining images of MIF in ischemic core and ischemic penumbra in I/R group and I/R + ISO-1 group (bar 50 μm). (**b**) Quantitative analysis of the expression of MIF by immunohistochemistry. (**c**) Western blot images of expression of MIF in peripheral ischemic brain tissue. (**d**) Quantification of MIF expression among the groups. *P < 0.05, **P < 0.01, ***P < 0.001.

Compared with core area in both I/R and I/R + ISO-1 groups, immunohistochemical staining showed higher expression of MIF in infarct penumbra (*F* = 22.97, *P* < 0.0001). In the penumbra, the expression of MIF in the I/R + ISO-1 group was lower than that in the I/R group (*P* = 0.0302). At the same time, we used Western blot to detect the expression of MIF in the cerebral ischemic penumbra, and the result showed that expression of MIF was different in different groups (*F* = 8.899, *P* = 0.0063). Compared with the I/R group, the expression of MIF decreased in the I/R + ISO-1 group (*P* = 0.0103) (Fig. 4).

### The MIF inhibitor ISO-1 reduces neuronal apoptosis

**Figure 5:**
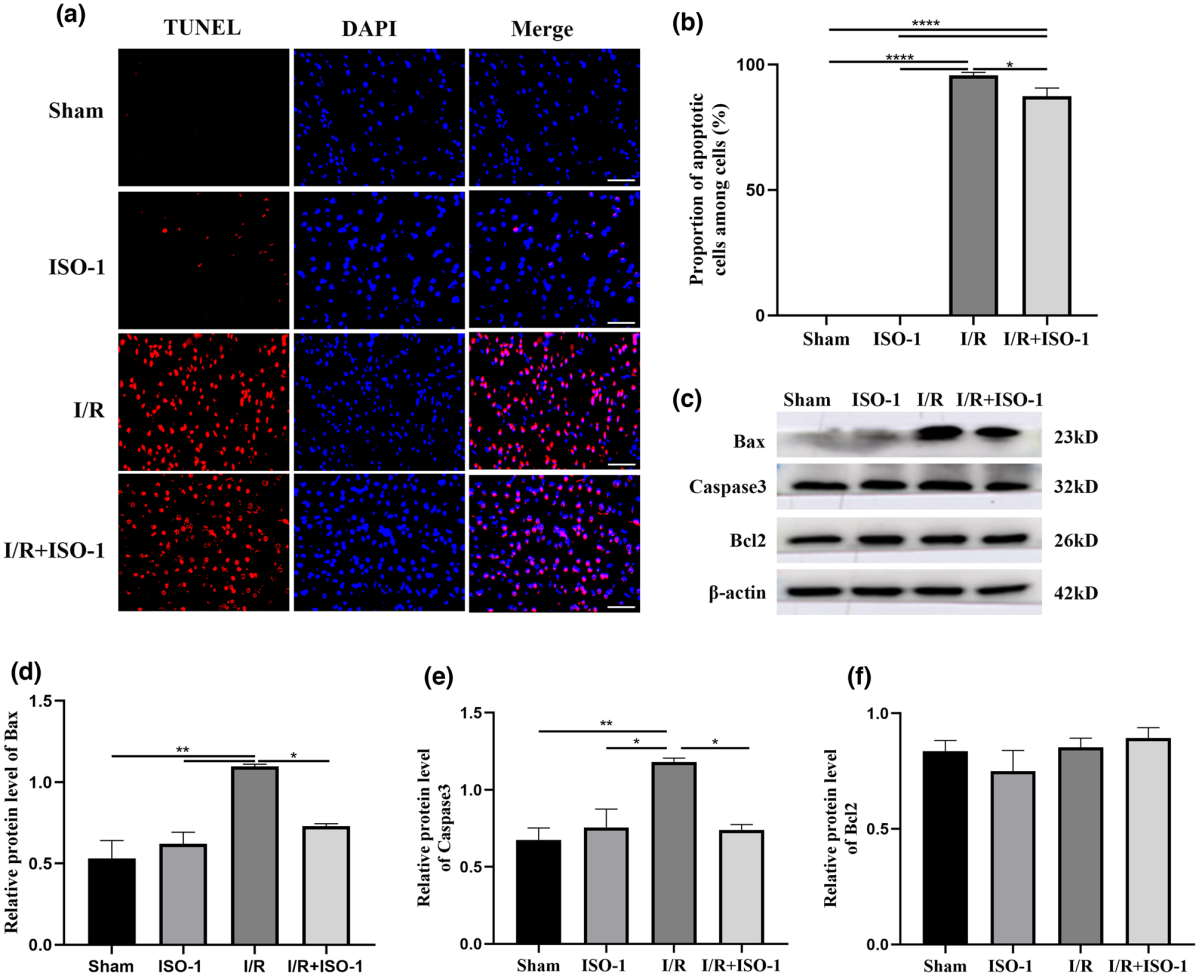
TUNEL staining analysis of apoptotic cells as well as the detection of apoptotic proteins. (**a**) Apoptotic cells were labeled with fluorescein isothiocyanate (red), and all nuclei were stained with DAPI (blue). Bar 50 μm. (**b**) Proportion of apoptotic cells in total cells. (**c**–**f**) Expression levels of Bax, caspase3, and Bcl2 protein were determined by Western blotting. *P < 0.05, **P < 0.01, ***P < 0.001, ****P < 0.0001.

The TUNEL results showed that there were few apoptotic cells in the Sham and ISO-1 groups, while there were more apoptotic cells in both I/R and the I/R + ISO-1 groups. The apoptosis rate of neurons in I/R + ISO-1 group was lower than that in I/R group (*P* = 0.0395). The results of western blot showed that there was no significant difference in the anti-apoptotic protein Bcl-2 among the groups (*F* = 1.064, *P* = 0.4169). The expressions of pro-apoptotic proteins Bax (*F* = 13.7, *P* = 0.0016) and caspase-3 (*F* = 9.56, *P* = 0.0051) were different among the groups. Compared with the sham group and ISO-1 group, the expression of Bax and caspase-3 increased in the I/R group (all *P* < 0.05). In comparison with the I/R group, the expressions of Bax and caspase-3 were significantly reduced (all *P* < 0.05) (Fig. 5).

### The MIF inhibitor ISO-1 increases the expression of the COX IV protein

**Figure 6:**
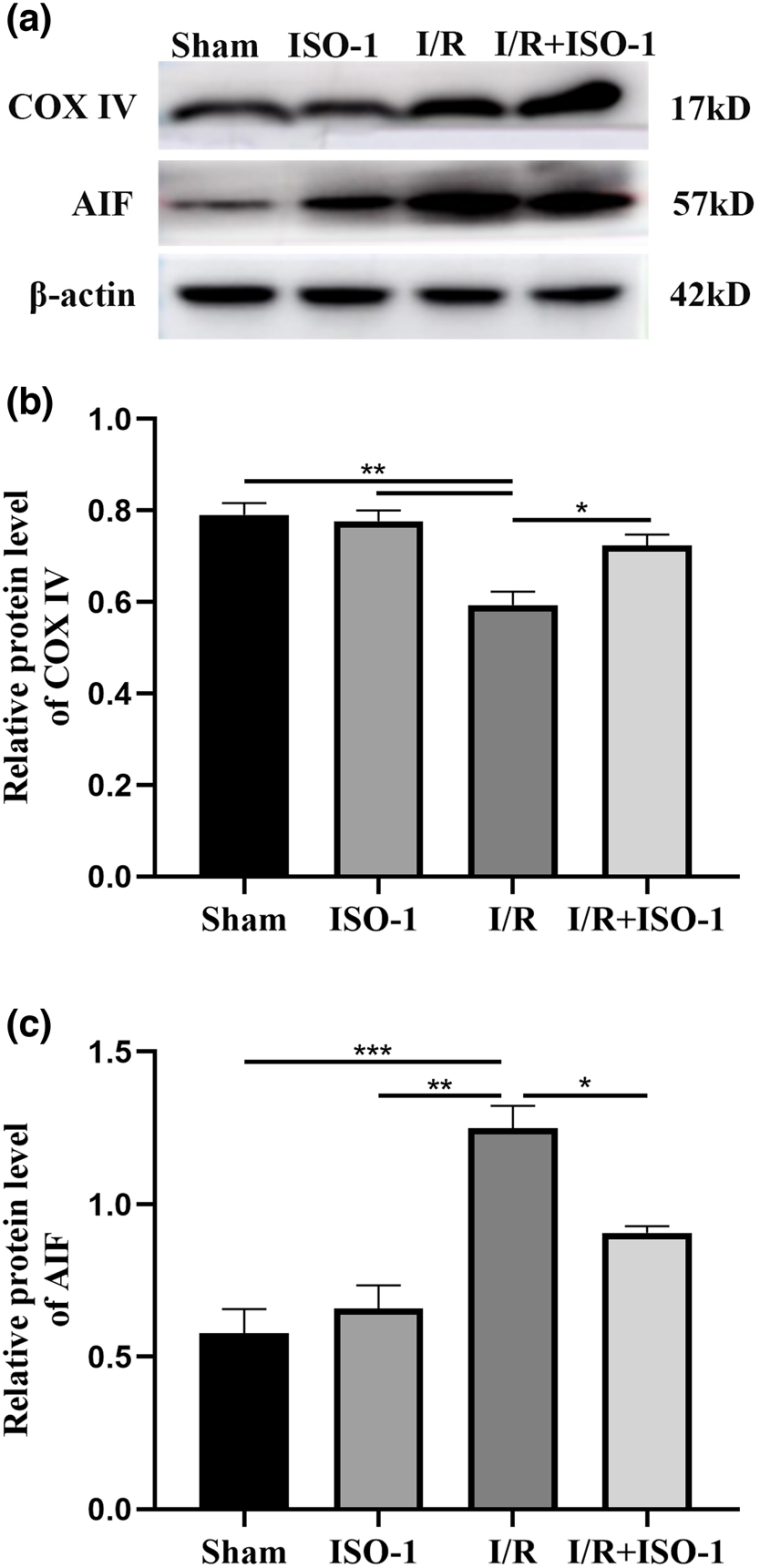
The expression levels of COX IV and AIF were detected by Western blotting. *P < 0.05, **P < 0.01, ***P < 0.001.

Western blotting showed that the expression of COX IV protein was different in sham group, ISO-1 group, I/R group and I/R + ISO-1 group (*F* = 12.08, *P* = 0.0024). The expression of COX IV protein in the I/R group and I/R + ISO-1 group was lower than that in the sham group and ISO-1 group (all *P* < 0.01). Compared with I/R group, the expression level of COX IV protein in I/R + ISO-1 group increased (*P* = 0.0303) (Fig. 6).

### MIF inhibitor ISO-1 reduces the expression of AIF protein

Western blotting showed that the expression of AIF protein in the I/R group and I/R + ISO-1 group was higher than that in the sham group and ISO-1 group (all *P* < 0.05). Compared with I/R group, the expression of AIF was lower in the I/R + ISO-1 group (*P* = 0.0277) (Fig. 6).

### MIF inhibitor ISO-1 inhibits nuclear translocation of AIF and EndoG in apoptotic cells

**Figure 7:**
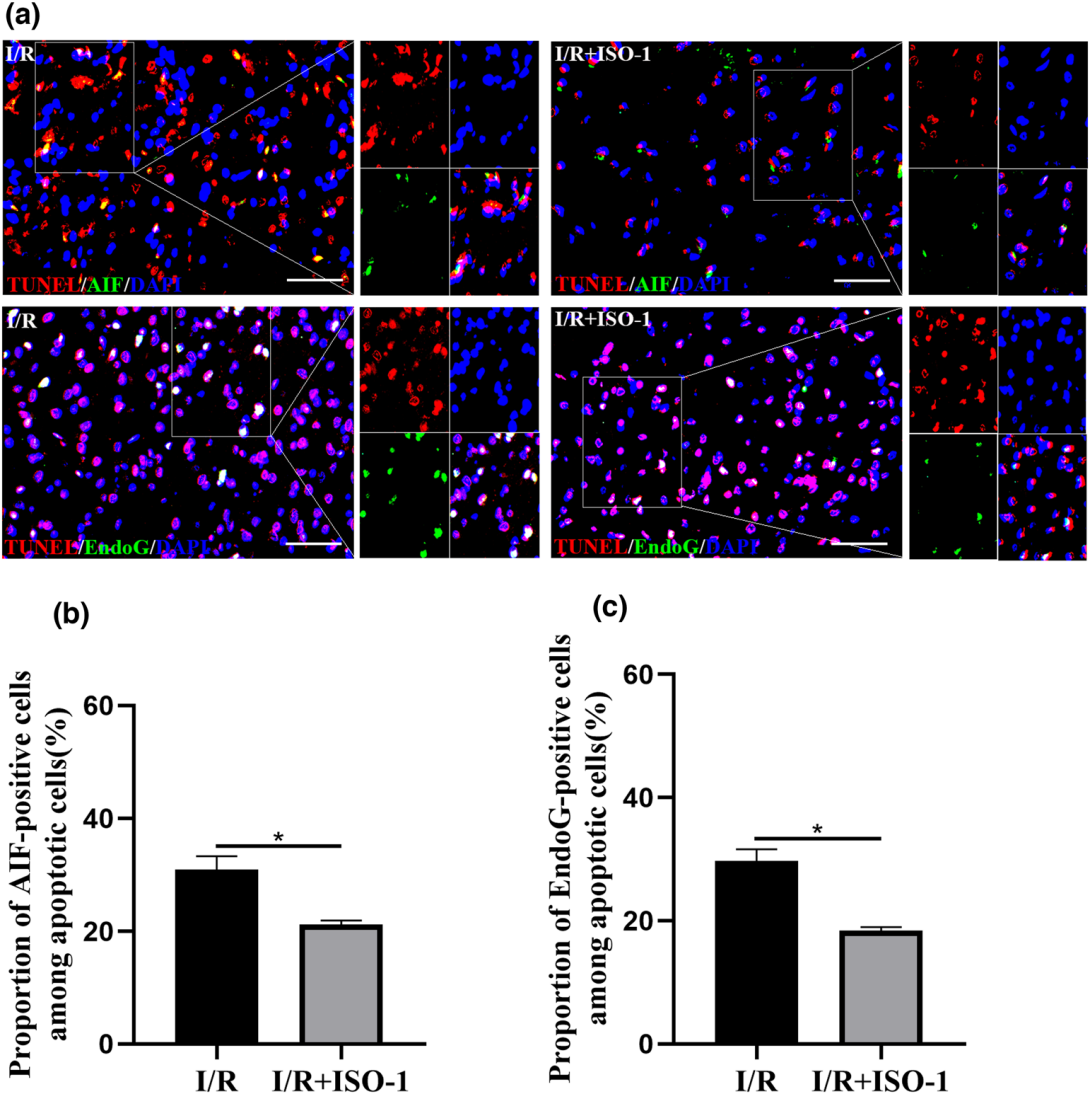
The colocalization of AIF and EndoG in apoptotic cells was detected by TUNEL and immunofluorescence staining. (**a**) Immunofluorescence staining of AIF (green), EndoG (green), DAPI (blue) and apoptotic cells (red). (**b**,**c**) The proportion of the number of TUNEL-positive cells showing the transfer of AIF and EndoG, which was from mitochondria to nucleus, to the total number of TUNEL-positive cells in different groups. *P < 0.05, bar 50 μm.

Immunofluorescence experiments showed there was a change of the positions of AIF and EndoG, which was from mitochondria to nucleus, in the ischemic penumbra after I/R. And the colocalization of AIF and EndoG in apoptotic cells was observed in the ischemic penumbra after ischemia–reperfusion. In comparison with I/R group, the proportion of the number of TUNEL-positive cells showing the transfer of AIF and EndoG, which was from mitochondria to nucleus, to the total number of TUNEL-positive cells was lower in the I/R + ISO-1 group (all *P* < 0.05, unpaired t-test) (Fig. 7).

**Discussion.**

MIF is a small molecular protein that can be expressed by many cells, and its molecular weight is 12.5 kD^21^. It played a role in infectious diseases and immune diseases by promoting the secretion of inflammatory factors, inhibiting apoptosis and regulating immunity^22^. In addition, the increase of MIF was also observed in hypothalamus and anterior pituitary, which also participated in some inflammatory reactions^23^. The role of MIF in ischemic stroke is complex. Previous studies have shown that MIF has a protective effect on neurons. For example, the lack of MIF may aggravate the brain damage after ischemia in female mice^24^. Su Hwan Bae et al.^25^ found that MIF could effectively induce the expression of BDNF and play a neuroprotective role in the hypoxic injury of neurons. Interestingly, the harmful effect of MIF on neurons has also been reported. For example, MIF knockout mice reduced neuronal death after ischemic stroke and promoted recovery of neurological function in mice^26^. In addition, administration of MIF antagonist ISO-1 might have neuroprotective effect^17^. Our previous experiments showed that the expression of MIF was the highest at 2 h of cerebral ischemia, so we chose reperfusion at 2 h of cerebral ischemia. In this study, after intraperitoneal injection of ISO-1, compared with I/R group, the expression of MIF in I/R + ISO-1 group decreased, which indicated that the drug was effective. We found that the expression of MIF after ischemia–reperfusion was higher than that in sham group, which was consistent with some previous research which indicated MIF was up-regulated in blood of hypoxia or stroke^17,27^.

(S,R)-3-(4-hydroxyphenyl)-4,5-dihydro-5-isoxazole methyl acetate (ISO-1) is an effective inhibitor of MIF tautomeric isomerase^28^. Studies have shown that MIF inhibitor ISO-1 can reduce ischemia-induced neuron damage and cerebral infarction in rats by reducing blood–brain barrier permeability and enhancing the tightness of brain endothelial cell connections^17^. After cerebral ischemia, a series of ischemic cascade reactions, such as apoptosis, inflammation and cytotoxicity would happen^29^, and apoptosis has a degree of reversibility. In the ischemic penumbra, apoptosis may occur after hours or days^1^. Therefore, saving dying cells of ischemic penumbra is the main purpose of clinical treatment of ischemic stroke. The results of our study showed that ISO-1 could reduce the expression of apoptotic proteins such as Bax, caspase3, and AIF in cerebral ischemic penumbra. In addition, we confirmed that ISO-1 could reduce neuronal apoptosis by TUNEL experiment. Interestingly, as mentioned above, MIF also has the effect of inhibiting apoptosis. After intraperitoneal injection of MIF inhibitor ISO-1 in rats, we observed the decrease of neuronal apoptosis, and the mechanism behind this phenomenon was worthy of further study.

The pathways leading to apoptosis are mainly classified into mitochondrial pathway, death receptor pathway and endoplasmic reticulum pathway^30^. Mitochondrion is a cystic structure composed of bilayer membrane and played the regulatory center role in the mitochondrial pathway. Its main function is producing energy through oxidative respiratory chain to maintain functions of cells^31^. As a subtype of COX, COX-IV, a protein complex located in the inner membrane of mitochondria, is a key enzyme for mitochondria to produce energy through oxidative respiratory chain^32^. Previous study has shown that the downregulation of COX-IV could lead to mitochondrial dysfunction and increase the level of mitochondrial reactive oxygen species^33^. Therefore, COX-IV and mitochondria coordinate with each other to maintain their respective functions. It’s of interest that COX-IV not only participate in mitochondrial ATP production, but also play an important role in cell apoptosis. It was reported that inhibiting COX-IV expression not only reduced ATP levels, but also made cells sensitive to apoptosis^34^. Another study showed that early up-regulation of COX-IV could protect neurons from mitochondrial oxidative damage^35^. Our study found that the expression of COX-IV in IR + ISO-1 group was significantly higher than that in I/R group. It was probably because that the intraperitoneal injection of ISO-1 reduced the sensitivity of neuronal apoptosis after cerebral ischemia, thereby reducing neuronal apoptosis.

After the interruption of cerebral blood flow, cell ischemia and hypoxia lead to mitochondrial dysfunction and membrane potential silencing^36,37^. When the function of mitochondria and membrane potential is damaged, cell death is inevitable^38^. After mitochondrial damage, an important step in inducing apoptosis is the transfer of AIF and EndoG from mitochondria to nucleus^39^. Oxidative stress is activated after the recovery of cerebral blood flow, resulting in abnormal mitochondrial structure and function, drastic reduction in energy supply, and cell death, an important mechanism of ischemia–reperfusion injury^40^. A study has shown that distal ischemic preconditioning could inhibit apoptosis through mitochondrial pathway^41^, thereby reducing cerebral ischemia–reperfusion injury. In this study, we observed that after ischemia–reperfusion, AIF in rat brain tissue was higher than that in sham group and the changes of the positions of AIF and EndoG, transporting from mitochondria to nucleus and locating in apoptotic cells. Compared with I/R group, the number of cells showing the transfer of AIF and EndoG, which was from mitochondria to nucleus, in I/R + ISO-1 group decreased. Therefore, we conclude that ISO-1 inhibits the release and transport of AIF and EndoG and reduces apoptosis mediated by endogenous mitochondrial pathway.

The role of MIF in ischemic stroke is complex. Therefore, the role of MIF inhibitor ISO-1 in ischemic stroke deserves further study.

### Summary

In conclusion, in the rat model of cerebral ischemia–reperfusion, MIF inhibitor ISO-1 can effectively improve the neurological deficit symptoms of ischemia–reperfusion injury and reduce the infarct volume. After cerebral ischemia, the structure and function of mitochondria were destroyed. AIF plays an important role in mitochondria-induced apoptosis. We speculate that ISO-1 inhibits mitochondrial pathway by reducing the transfer of AIF and EndoG from mitochondria to nucleus, which improves the resistance of nerve cells to ischemia and reduces the apoptosis of neurons, thereby realizing the neuroprotective effect (Supplementary Information).

## Supplementary Information

Supplementary Information.

## Data Availability

All data for this study can be obtained in writing from the corresponding author.

## References

[1] Radak D (2017). Apoptosis and acute brain ischemia in ischemic stroke. Curr. Vasc. Pharmacol..

[2] Go AS (2014). Heart disease and stroke statistics–2014 update: A report From the American Heart Association. Circulation.

[3] Cheng X, Zhang F, Li J, Wang G (2019). Galuteolin attenuates cerebral ischemia/reperfusion injury in rats via anti-apoptotic, anti-oxidant, and anti-inflammatory mechanisms. Neuropsychiatr. Dis. Treat..

[4] Iadecola C, Anrather J (2011). The immunology of stroke: From mechanisms to translation. Nat. Med..

[5] Bredesen DE, Rao RV, Mehlen P (2006). Cell death in the nervous system. Nature.

[6] Amani H (2019). Would colloidal gold nanocarriers present an effective diagnosis or treatment for ischemic stroke?. Int. J. Nanomed..

[7] Li WH (2020). Baicalein attenuates caspase-independent cells death via inhibiting PARP-1 activation and AIF nuclear translocation in cerebral ischemia/reperfusion rats. Apoptosis.

[8] Cheng A, Hou Y, Mattson MP (2010). Mitochondria and neuroplasticity. ASN Neuro.

[9] Dawson TM, Dawson VL (2017). Mitochondrial mechanisms of neuronal cell death: Potential therapeutics. Annu. Rev. Pharmacol. Toxicol..

[10] Kim Y (2018). Early treatment with poly(ADP-ribose) polymerase-1 inhibitor (JPI-289) reduces infarct volume and improves long-term behavior in an animal model of ischemic stroke. Mol. Neurobiol..

[11] Guardia-Laguarta C (2019). PINK1 content in mitochondria is regulated by ER-associated degradation. J. Neurosci..

[12] Sun C (2004). Macrophage migration inhibitory factor: An intracellular inhibitor of angiotensin II-induced increases in neuronal activity. J. Neurosci..

[13] Wang L (2009). Upregulation of macrophage migration inhibitory factor gene expression in stroke. Stroke.

[14] Inácio AR, Bucala R, Deierborg T (2011). Lack of macrophage migration inhibitory factor in mice does not affect hallmarks of the inflammatory/immune response during the first week after stroke. J. Neuroinflamm..

[15] Nasiri E (2020). Key role of MIF-related neuroinflammation in neurodegeneration and cognitive impairment in Alzheimer's disease. Mol. Med..

[16] Zhao Y (2020). Inhibition of macrophage migration inhibitory factor protects against inflammation through a toll-like receptor-related pathway after diffuse axonal injury in rats. Biomed. Res. Int..

[17] Liu YC (2018). Cytokine MIF enhances blood-brain barrier permeability: Impact for therapy in ischemic stroke. Sci. Rep..

[18] Yang Y (2022). Geraniin protects against cerebral ischemia/reperfusion injury by suppressing oxidative stress and neuronal apoptosis via regulation of the Nrf2/HO-1 pathway. Oxid. Med. Cell. Longev..

[19] Chen J (2001). Therapeutic benefit of intracerebral transplantation of bone marrow stromal cells after cerebral ischemia in rats. J. Neurol. Sci..

[20] Ashwal S, Tone B, Tian HR, Cole DJ, Pearce WJ (1998). Core and penumbral nitric oxide synthase activity during cerebral ischemia and reperfusion. Stroke.

[21] Bernhagen J, Calandra T, Cerami A, Bucala R (1994). Macrophage migration inhibitory factor is a neuroendocrine mediator of endotoxaemia. Trends Microbiol..

[22] Wang SS, Cen X, Liang XH, Tang YL (2017). Macrophage migration inhibitory factor: A potential driver and biomarker for head and neck squamous cell carcinoma. Oncotarget.

[23] Kindt N, Journe F, Laurent G, Saussez S (2016). Involvement of macrophage migration inhibitory factor in cancer and novel therapeutic targets. Oncol. Lett..

[24] Turtzo LC (2013). Deletion of macrophage migration inhibitory factor worsens stroke outcome in female mice. Neurobiol. Dis..

[25] Bae SH (2020). Brain-derived neurotrophic factor mediates macrophage migration inhibitory factor to protect neurons against oxygen-glucose deprivation. Neural Regen. Res..

[26] Inácio AR, Ruscher K, Leng L, Bucala R, Deierborg T (2011). Macrophage migration inhibitory factor promotes cell death and aggravates neurologic deficits after experimental stroke. J. Cereb. Blood Flow Metab..

[27] Zis O, Zhang S, Dorovini-Zis K, Wang L, Song W (2015). Hypoxia signaling regulates macrophage migration inhibitory factor (MIF) expression in stroke. Mol. Neurobiol..

[28] Al-Abed Y (2005). ISO-1 binding to the tautomerase active site of MIF inhibits its pro-inflammatory activity and increases survival in severe sepsis. J. Biol. Chem..

[29] Brouns R, De Deyn PP (2009). The complexity of neurobiological processes in acute ischemic stroke. Clin. Neurol. Neurosurg..

[30] Hu H, Tian M, Ding C, Yu S (2018). The C/EBP homologous protein (CHOP) transcription factor functions in endoplasmic reticulum stress-induced apoptosis and microbial infection. Front. Immunol..

[31] Spinelli JB, Haigis MC (2018). The multifaceted contributions of mitochondria to cellular metabolism. Nat. Cell Biol..

[32] Fukuda R (2007). HIF-1 regulates cytochrome oxidase subunits to optimize efficiency of respiration in hypoxic cells. Cell.

[33] Geng SL, Zhang XS, Xu WH (2021). COXIV and SIRT2-mediated G6PD deacetylation modulate ROS homeostasis to extend pupal lifespan. Febs J..

[34] Li Y, Park JS, Deng JH, Bai Y (2006). Cytochrome C oxidase subunit IV is essential for assembly and respiratory function of the enzyme complex. J. Bioenerg. Biomembr..

[35] Hao YH (2018). HIF-1α regulates COXIV subunits, a potential mechanism of self-protective response to microwave induced mitochondrial damages in neurons. Sci. Rep..

[36] Broughton BR, Reutens DC, Sobey CG (2009). Apoptotic mechanisms after cerebral ischemia. Stroke.

[37] Lo EH, Moskowitz MA, Jacobs TP (2005). Exciting, radical, suicidal: How brain cells die after stroke. Stroke.

[38] Zhang J (2017). A novel ADOA-associated OPA1 mutation alters the mitochondrial function, membrane potential, ROS production and apoptosis. Sci. Rep..

[39] Hu WL (2017). Bid-induced release of AIF/EndoG from mitochondria causes apoptosis of macrophages during infection with leptospira interrogans. Front. Cell. Infect. Microbiol..

[40] Suliman HB, Piantadosi CA (2016). Mitochondrial quality control as a therapeutic target. Pharmacol. Rev..

[41] Lv J (2020). RIPC provides neuroprotection against ischemic stroke by suppressing apoptosis via the mitochondrial pathway. Sci. Rep..

