# Supplementary material for: Ischemic stroke protected by ISO-1 inhibition of apoptosis via mitochondrial pathway
Source: Sci Rep. 2023 Feb 16;13:2788. doi: 10.1038/s41598-023-29907-z (PMC9935850; doi:10.1038/s41598-023-29907-z)

# Ischemic stroke protected by ISO-1 inhibition of apoptosis via mitochondrial pathway

Wanli Ji[1,2], Yaoxin Ren[1,2], Xiaolian Wei[1], Xiangxiang Ding[1,2], Yihan Dong[1], Bin Yuan[1,2*]

・Thank you for your valuable comments on our article.

・In order to obtain better gel images, we cut the PVDF membrane before incubating the antibody and only the part of the target proteins was retained. At the request of the journal, we have tried our best to provide all pictures of Western blot performed on the same PVDF membrane.

1. The following pictures are from the same PVDF membrane.

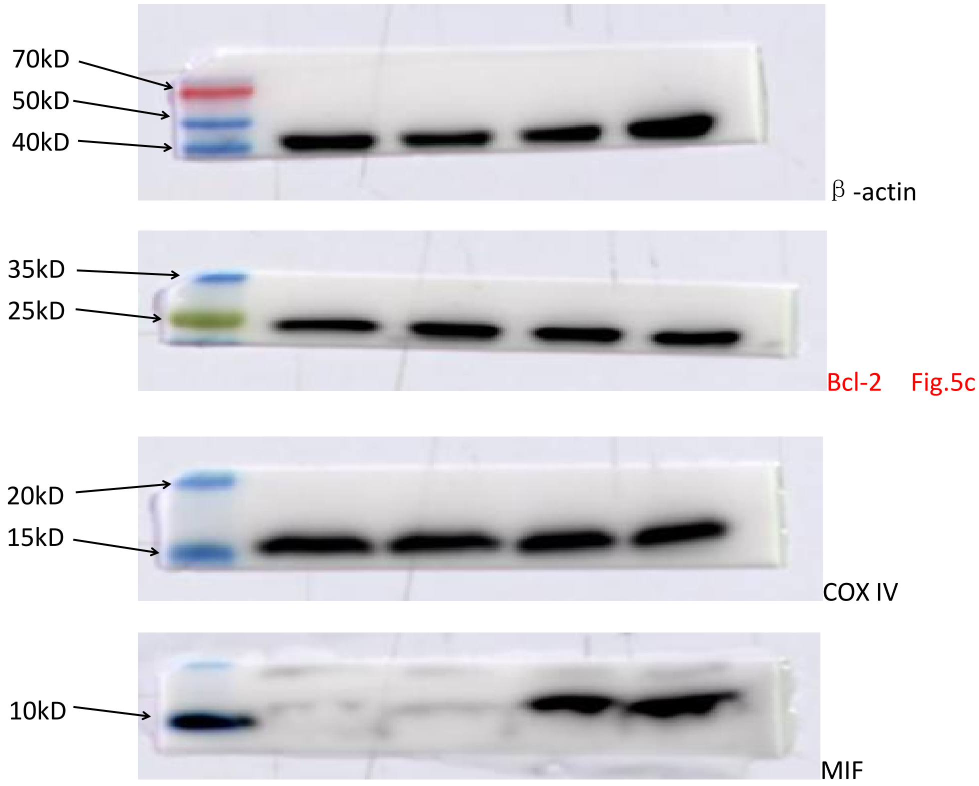


2. The following pictures are from the same PVDF membrane.

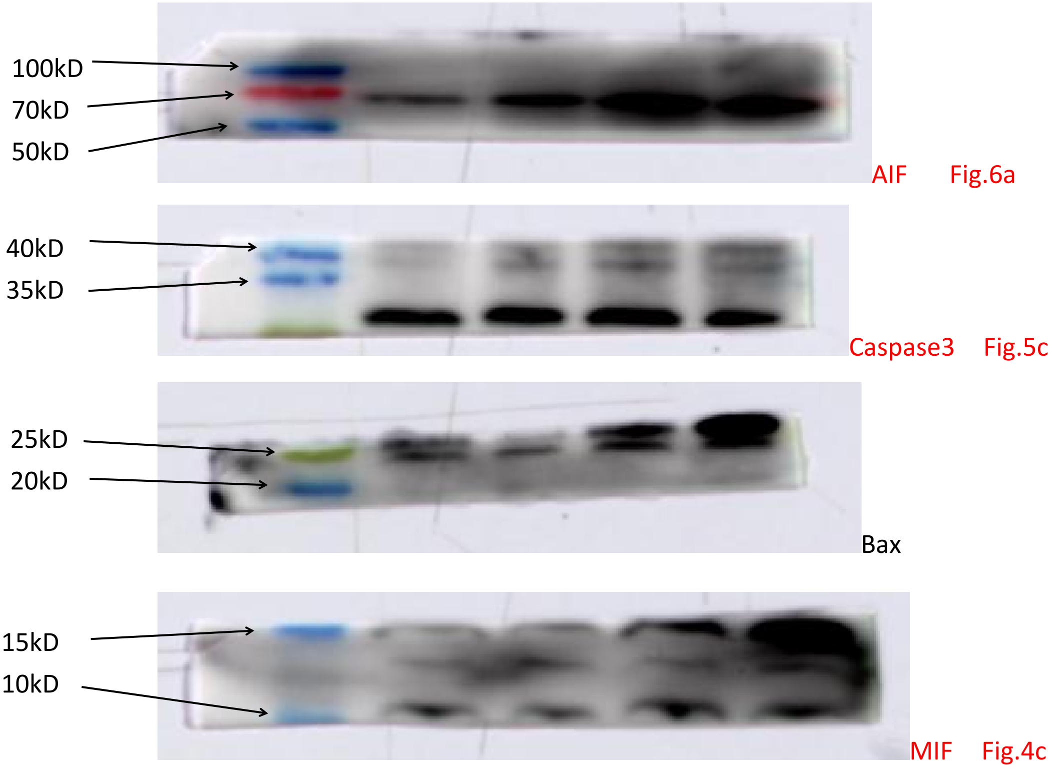

3. The following pictures are from the same PVDF membrane.

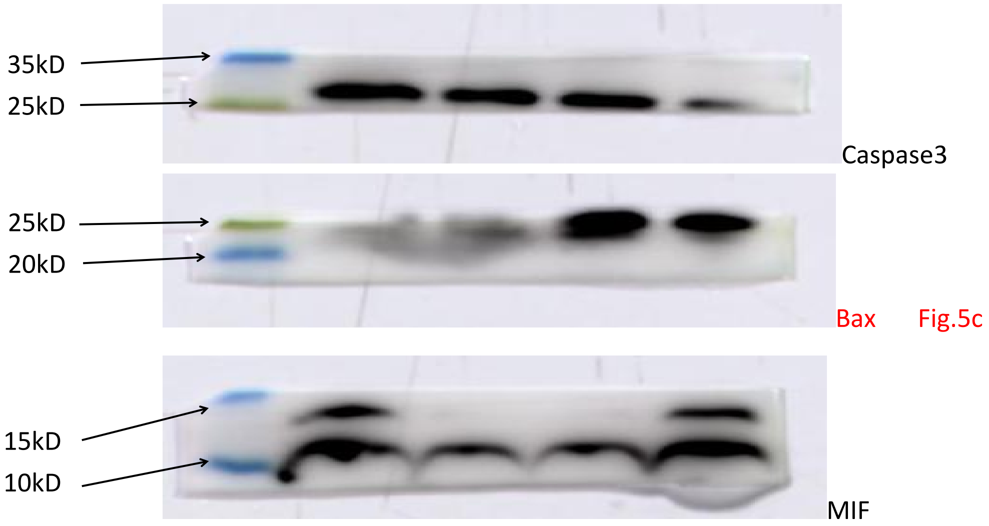


4. The following pictures are from the same PVDF membrane.

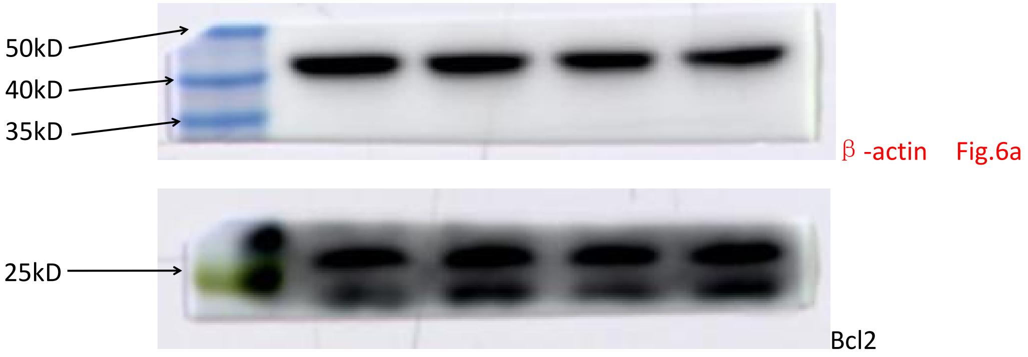


5. The following pictures are from the same PVDF membrane.

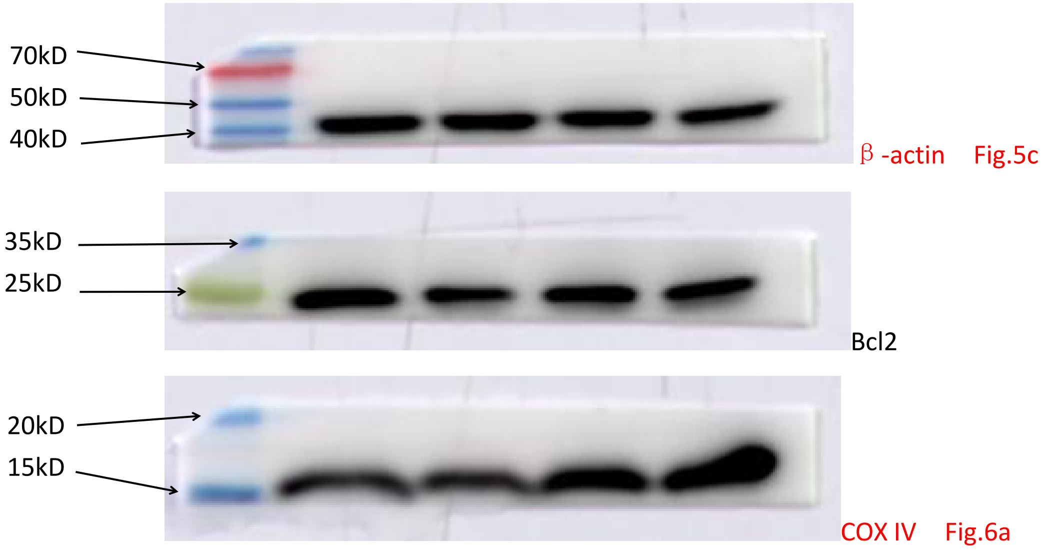

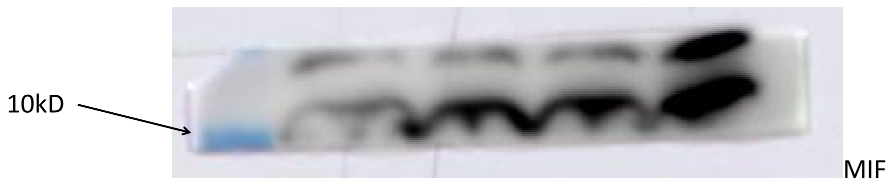


6. The following pictures are from the same PVDF membrane.

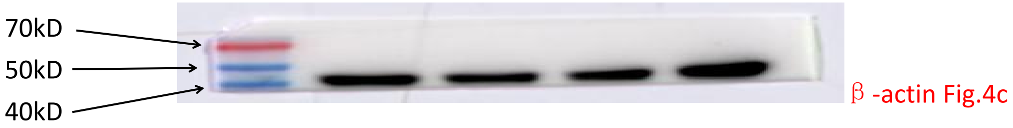


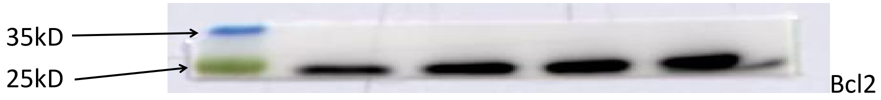


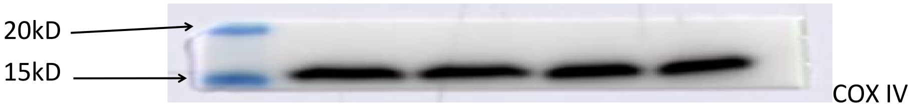


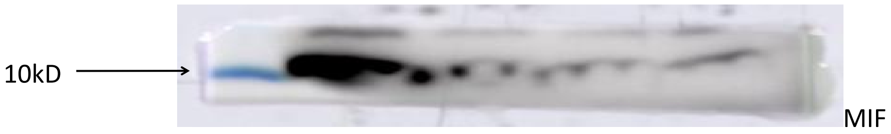

Supplement: Supplementary file 1 — Supplementary Information. [file 41598_2023_29907_MOESM1_ESM.pdf]
